# Diagnostic Accuracy and Safety of Coaxial System in Oncology Patients Treated in a Specialist Cancer Center With Prospective Validation Within Clinical Trial Data

**DOI:** 10.3389/fonc.2020.01634

**Published:** 2020-09-04

**Authors:** Khurum Khan, Reyes Gonzalez-Exposito, David Cunningham, Dow-Mu Koh, Andrew Woolston, Louise Barber, Beatrice Griffiths, Kyriakos Kouvelakis, Vanessa Calamai, Monia Bali, Nasir Khan, Annette Bryant, Claire Saffery, Charles Dearman, Ruwaida Begum, Sheela Rao, Naureen Starling, David Watkins, Ian Chau, Chiara Braconi, Nicola Valeri, Marco Gerlinger, Nicos Fotiadis

**Affiliations:** ^1^Department of Gastrointestinal Oncology, UCL Cancer Institute, University College NHS Foundation Trust, London, United Kingdom; ^2^Department of Medicine, The Royal Marsden NHS Trust, London, United Kingdom; ^3^Cancer Research UK Cancer Imaging Centre, Division of Radiotherapy and Imaging, The Institute of Cancer Research and Royal Marsden Hospital, London, United Kingdom; ^4^Translational Oncogenomics Laboratory, Centre for Evolution and Cancer, The Institute of Cancer Research, London, United Kingdom; ^5^Division of Molecular Pathology, The Institute of Cancer Research, London, United Kingdom

**Keywords:** coaxial core-needle biopsy system, tissue biopsies, formalin-fixed paraffin-embedded, clinical trials, genomic analysis

## Abstract

**Background:**

Image-guided tissue biopsies are critically important in the diagnosis and management of cancer patients. High-yield samples are also vital for biomarker and resistance mechanism discovery through molecular/genomic analyses.

**Patients and Methods:**

All consecutive patients who underwent plugged image-guided biopsy at Royal Marsden from June 2013 until September 2016 were included in the analysis. In the next step, a second cohort of patients prospectively treated within two clinical trials (PROSPECT-C and PROSPECT-R) were assessed for the DNA yield from biopsies assessed for complex genomic analysis.

**Results:**

A total of 522 plugged core biopsies were performed in 457 patients [men, 52%; median age, 63 years (range, 17–93)]. Histological diagnosis was achieved in 501 of 522 (96%) performed biopsies. Age, gender, modality, metastatic site, and seniority of the interventionist were not found to be significant factors associated with odds of failure on a logistic regression. Seventeen (3.3%) were admitted due to biopsy-related complications; nine, three, two, one, one, and one were admitted for grade I/II pain control, sepsis, vasovagal syncope, thrombosis, hematuria, and deranged liver functions, respectively; two patients with right upper quadrant pain after liver biopsy were found to have radiologically confirmed subcapsular hematoma requiring conservative treatment. One patient (0.2%) developed grade III hemorrhage following biopsy of a gastric gastrointestinal stromal tumor (GIST). Overall molecular analysis was successful in 89% (197/222 biopsies). Prospective validation in 62 biopsies gave success rates of 92.06 and 79.03% for DNA extraction of >1 μm and tmour content of >20%, respectively.

**Conclusion:**

The probability of diagnostic success for complex molecular analysis is increased with plugged large coaxial needle biopsy technique, which also minimizes complications and reduces hospital stay. High-yield DNA acquisition allows genomic molecular characterization for personalized medicine.

## Introduction

While cancer management and treatment options have significantly improved during the last few years, our knowledge and understanding about mechanisms of response, and/or resistance to anticancer therapies remain relatively sparse. To date, this relative lack of understanding is partially due to difficulties in accessing prospectively collected tissue and blood samples from systemic anticancer therapy (SACT)-resistant tumors.

Image-guided tissue biopsies are not just important in establishing an accurate histopathological diagnosis and standard cancer management; high-yield samples are also vital in understanding the molecular and genomic characteristics of tumors. Genomic analyses on tumor samples broadly fall into two categories including (1) targeted approaches investigating a limited number of genes that are known to influence clinical decision making and (2) whole exome or genome sequencing frequently adopted in exploratory research studies to learn about new mechanisms of response or resistance to SACT (1, 2). Conventional formalin-fixed paraffin-embedded (FFPE) samples obtained during diagnostic procedures may not be sufficient for such analyses to be realized. For instance, the data from The Cancer Genome Atlas (TCGA) studies showed that fresh frozen material from primary tumor resection specimens was associated with a tumor content of 60% (3). Moreover, using FFPE DNA for large-scale genomic studies may demonstrate mutations that have occurred as a result of the fixation process, which makes it difficult to distinguish real tumor variants from these fixation artifacts. Furthermore, low-quality fragmented DNA can fail quality control in the preanalytical stage impairing success rates.

While a number of retrospective studies have demonstrated the safety and accuracy of diagnostic biopsies (4–6), data interpretation from such studies has often been hampered by small numbers, the lack of information on yield for molecular/genomic characterization of tumors, and the lack of prospective validation. At Royal Marsden (RM), we have been using coaxial core-needle biopsy (CNB) system and a preformed gelatin sponge sealing device to conduct solid organ core biopsies in order to minimize the number of passes and reduce the risk of complications, respectively. We present here the largest dataset demonstrating the safety and accuracy of this approach. Moreover, we took the opportunity to utilize a cohort of patients from two prospective clinical trials to validate tumor yields from biopsies in these translational studies.

## Materials and Methods

### Study Design

All consecutive patients who underwent plugged image-guided biopsy at RM from June 2013 until September 2016 were included in the analysis. Data including gender, age, primary tumor, biopsy site, needle gage, interval between biopsy and discharge, incidence of complications, and biopsy success were collected. The study was approved by the RM Institutional review board.

### Biopsy Technique

**FIGURE 1:**
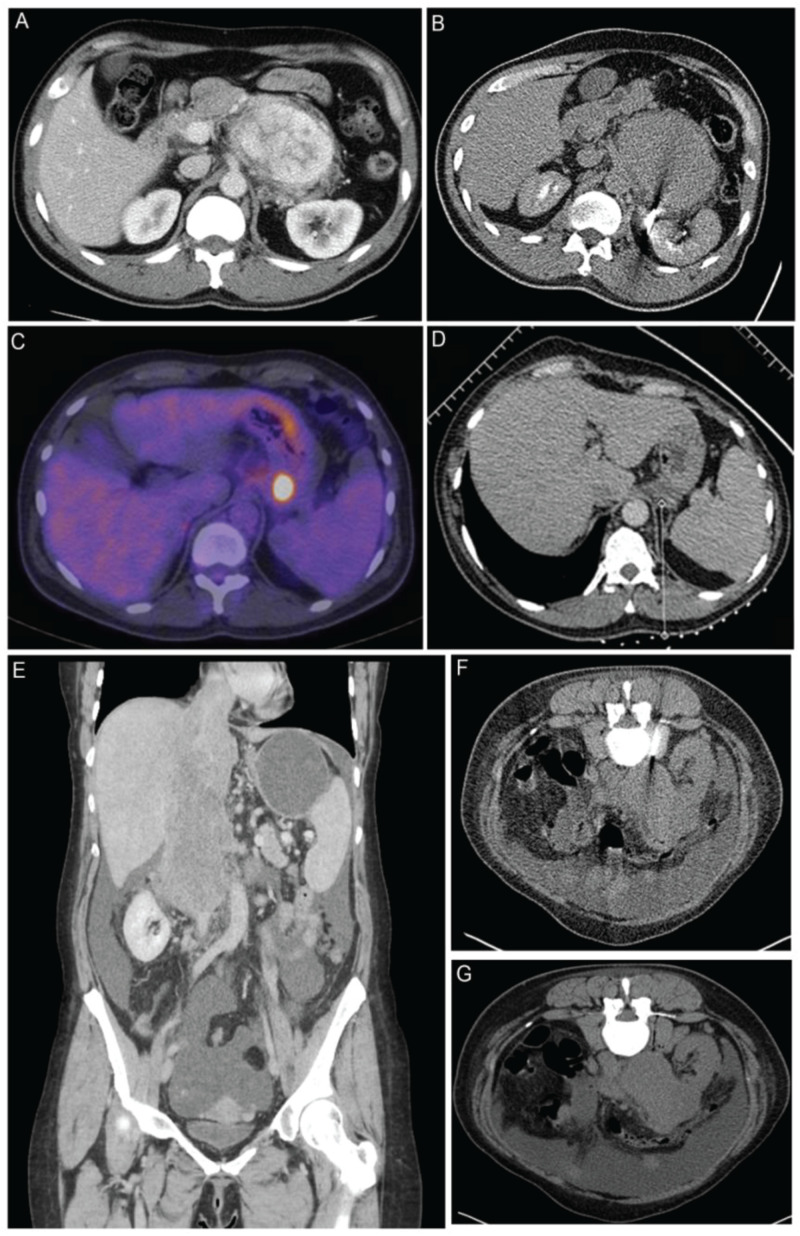
Biopsy examples of patients within the study **(A)** A computed tomography (CT) of a patient with a highly vascular retroperitoneal mass thought to be too high risk to biopsy at the local hospital. Surgery was also considered to be high risk of R1/R2 resection, and a CT-guided biopsy was recommended by our multidisciplinary team (MDT). **(B)** Biopsy was performed with a 15-G/16-G coaxial needle. The tract was plugged with 16-G Hunter plugs, and there were no complications. The biopsy showed an inflammatory myofibroblastic tumor, which responded well on steroids and an operation was avoided. **(C)** PET/CT of a 57-year-old patient with relapsed Hodgkin’s lymphoma after six cycles of ABVD chemotherapy. There was response in all sites of disease with the exception of a plaque of tissue behind the fundus of the stomach, which appear [18F]-fluorodeoxyglucose (FDG) avid on PET scan. A decision of the MDT was made to biopsy the lesion in order to exclude transformation of lymphoma. **(D)** The 17-G coaxial needle was placed medial to the left adrenal and above the splenic vessels adjacent to the lesion. Three cores were taken, and the tract was plugged. There were no complications. The biopsy showed Hodgkin’s lymphoma, which responded well to systemic therapy and consolidation RT. **(E)** Coronal CT images of a 48-year-old patient with a large tumor of the inferior vena cava (IVC) extending from the level of the renal veins to the right atrium. Occluded hepatic veins and ascites can be seen on the scan. **(F)** The lesion was biopsied with a 15-G/16-G coaxial needle. **(G)** The tract was plugged with three gelfoam pledgets. A diagnosis of leiomyosarcoma of the IVC was made, and the procedure had no complications.

The biopsies were performed by a Consultant Interventional Radiologist (IR) or an IR fellow under supervision. Ultrasound and CT guidance was used based on the location of the lesion. Conscious sedation was administered along with local anesthesia, when required, to maximize cooperation and improve patient experience (Figure 1). A 15- or 17-G coaxial needle was inserted under direct image guidance at the edge of the lesion and two to six cores were obtained with a 16- or 18-G automatic core-biopsy needle (True-Core II, Argon Medical Devices, Frisco, TX, United States), respectively. Different areas inside the lesion were sampled by changing the angle and position of the coaxial needle. After the samples were collected, one to four preformed 16- or 18-G gelatin foam pledgets (Hunter biopsy-sealing device, Vascular Solutions Inc., Minneapolis, MN, United States) were deployed through the coaxial along the tract of the needle to facilitate hemostasis. The gelatin resorbs completely within 12 weeks.

### Validation Cohort

In the second step, a validation cohort of patients prospectively treated within two clinical trials was used to assess the DNA yield utilized for genomic analysis. The two trials included *PROSPECT-C* [clinical trials.gov number (NCT02994888)] (2, 7) and *PROSPECT-R* [clinical trials.gov number (NCT03010722)] (1); phase II, open label, non-randomized studies of antiepidermal growth factor receptor (anti-EGFR) monoclonal antibodies and regorafenib in patients with *RAS* wild type and *RAS* mutant refractory metastatic colorectal cancer (CRC), respectively. All participants in both studies were required to have mandatory pretreatment biopsies (6 cores), biopsies at partial response in *PROSPECT-C* and stable disease at 2 months in *PROSPECT-R* (6 cores), and at the time of progression (6–12 cores from two suitable progressing metastatic sites).

### Prospective Tissue Collection Procedures

Fresh frozen and FFPE tissue samples were obtained, and plasma collection was conducted as per the study protocols at the clinically relevant defined time points. Sixteen-gage core biopsy was used to collect three or four fresh biopsy specimens and one or two specimens fixed in formalin and paraffin embedded. Within the trials, approximately 25% of the total length of a core was detached for primary culture, and the remaining ∼75% of the core was snap frozen and used for genomic analysis. One core was transported to establish tumor-derived organoids and targeted panel validation (8). One core was used for genomic analysis after being placed into cryovials and immediately snap frozen in liquid nitrogen. The remaining two cores were placed straight into formalin and embedded in paraffin wax. Primary morphological and immunohistochemical analysis was performed by the histopathologist on the FFPE specimen for confirmation of diagnosis. The samples were then stored in the GI and Lymphoma Research Bank of the RM, anonymized by trial number and time point.

### Tissue Sample Processing

**FIGURE 2:**
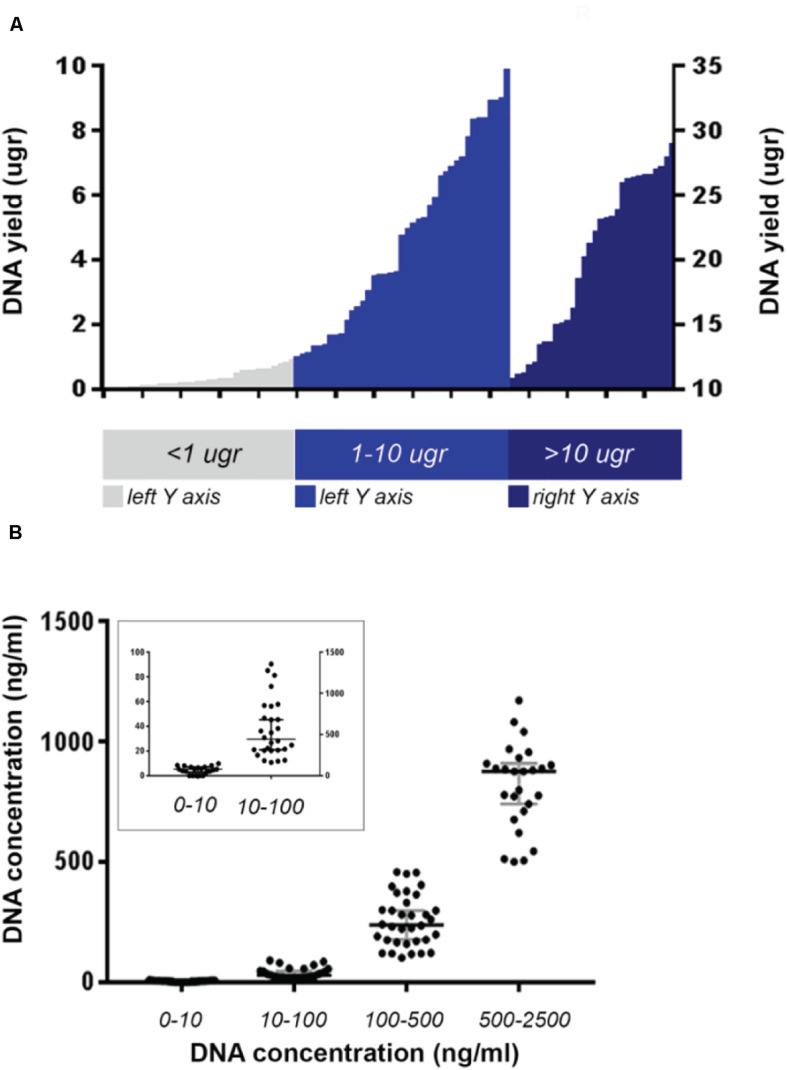
Maximum DNA yield of the whole analyzed cohort from PROSPECT-C and PROSPECT-R patients. **(A)** Cases with DNA yield >10 μg are plotted against right *Y*-axis. **(B)** Cases divided according to their DNA concentration. Median value and with 95% CI represented in gray bars. In the small square cases with DNA concentration < 100 ng/ml are plotted against the left *Y*-axis.

Biopsy cores were snap frozen in liquid nitrogen at the time of collection. Genomic gDNA and mRNA were co-extracted from cores using the Qiagen All-Prep kit. DNA was also isolated from whole blood samples using the Qiagen QIAamp DNA Blood Mini kit (Figure 2).

### Whole Exome Sequencing

A minimum of 500 ng of gDNA was prepared for whole exome sequencing (WES) using the Agilent SureSelect Human All Exon v5 capture library, according to the manufacturers’ protocol. The resulting libraries were sequenced to a mean depth of 100× using paired-end 100 reads on an Illumina HiSeq 2500. High-quality reads were aligned to the National Center for Biotechnology information (NCBI) reference genome (hg19) using BWA (v0.7.12) and SAMtools (v0.1.19) to remove duplicates. Tumor content was estimated based on the CNVkit (v0.8.1) copy number profile.

### Sanger Sequencing

**FIGURE 3:**
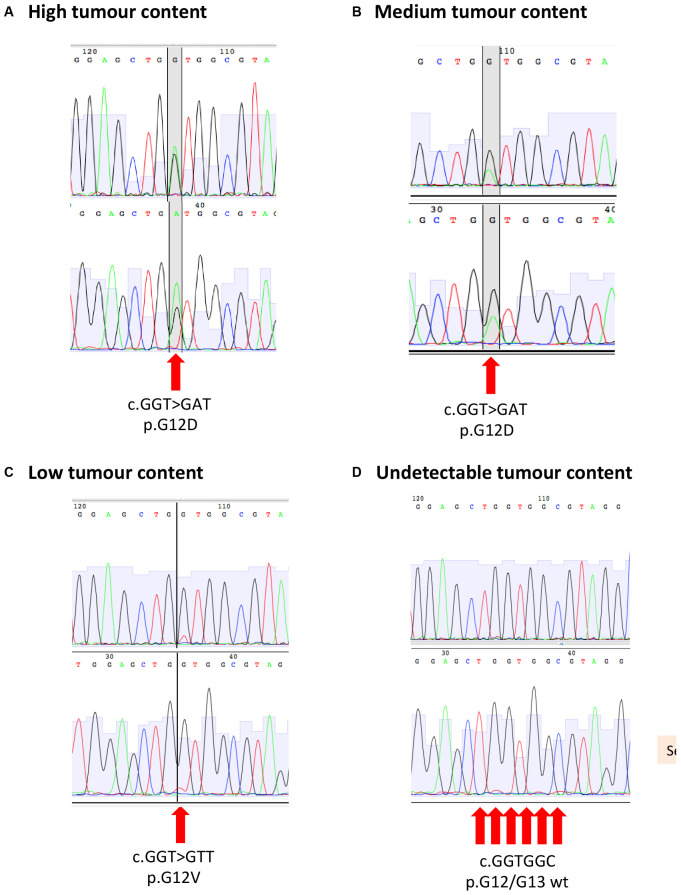
Sanger sequencing for KRAS p.G12/G13 mutation with example of **(A)** high tumour content, **(B)** medium tumour content, **(C)** low tumour content, and **(D)** undetectable tumour content.

For patients with a known tumour variant, PCR was performed on 20 nanograms of gDNA using M13F/R-tailed mutation specific primers (Life Technologies; Supplementary Table 1) and Q5 High-Fidelity 2 × Master Mix (NEB) on an Eppendorf Mastercycler Nexus GSX1. Primer-specific annealing temperatures for Q5 polymerase were established using the NEB online Tm calculator. PCR products were cleaned using Qiaquick PCR purification kit (Qiagen), and 15 ng DNA was submitted for M13F and M13R sequencing using the Mix2Seq service (Eurofins Genomics). Ab1 traces were visualized and compared to the reference sequence using ApE software^1^. Sample tumour content was estimated from the relative abundance of wild-type and variant peaks (Figure 3 and Supplementary Table 1).

### Statistical Design

The success rate of biopsies was determined by the ability to perform standard molecular testing on tissue specimens and safety determined by frequency of complications and extended hospital stay. Encrypted data were collected in a password-protected Excel file and statistical analysis performed using STATA13. Chi-squared analysis was undertaken to identify baseline characteristics that provided independent association with failure and success rates.

## Results

### Overall Safety of Image-Guided Biopsies and Cox Regression Analysis

A total of 522 tissue biopsies were performed in 457 patients [men, 48%; median age, 63 years (range, 23–86)] (Supplementary Table 2). Two, three, and four biopsies were obtained from 51 (11.2%), 13 (2.8%), and 1 (0.2%) patients, respectively, at different time points as part of clinical trial protocols. Histological diagnosis was achieved in 501 of 522 (96%) performed biopsies. Same-day discharge was achieved for 444 (85.1%) procedures as outpatients, 35 (6.7%) and 17 (3.3%) had planned inpatient and elective procedures, respectively, and 8 (1.5%) patients were kept in for overnight observation after a late evening procedure. Seventeen (3.3%) were admitted with the following biopsy-related complications: grade I/II pain control (nine), sepsis (one), vasovagal syncope (two), thrombosis (one), hematuria (one), and deranged liver functions (one). Two patients with right upper quadrant pain had radiologically confirmed subcapsular hematoma requiring conservative treatment. One patient (0.2%) developed grade III hemorrhage requiring transfusion of 2 U of packed red blood cells following biopsy of a gastric gastrointestinal stromal tumor (GIST). In 21 of 522 biopsies, diagnosis was not achieved due to sampling error during needle placement. These were small lesions not well visualized with ultrasound and CT, and normal tissue adjacent to the lesion was consequently biopsied. When patients were divided into two groups including those who underwent “liver biopsy” (*n* = 284 biopsies from 231 patients) and all other biopsies except liver, i.e., “others” (*n* = 238 biopsies from 228 patients). Success rates of 95.02 and 98.32% were observed in the two groups, respectively (Supplementary Tables 3A,B).

### Chi-Squared Tests to Assess Covariates of Failure

Results from chi-squared tests showed that the covariates of age category at earliest biopsy date, gender, modality of image guidance, metastatic site, and seniority of the interventionist were not associated with the occurrence of failure. Association of site of biopsy (others vs. liver), however, showed a significant trend in favor of other organs vs. liver, although the difference was not found to be numerically and clinically of significant impact (*p* = 0.053). Patients who had biopsy within clinical trials (*n* = 163 biopsies) vs. those who underwent routine clinical diagnostic biopsies (*n* = 338) showed a success rate of 98.79 and 95.48%, respectively. Chi-squared test demonstrated significance in favor of patients treated within clinical trials (*p* = 0.07; Supplementary Table 4).

### Validated Genomic Testing in Patients With Metastatic Colorectal Cancer

Given that metastatic colorectal cancer (mCRC) patients underwent genomic profiling for clinically actionable mutations such as *KRAS*, *NRAS*, and *BRAF* analysis routinely with a clinically validated COBAS panel, we rationalized separating this cohort from the remaining patients. Of the total 144 patients with mCRC, 17 repeat charts and 38 patients who were referred from other hospitals were excluded. Of the remaining 89 patients, 2 (2.25%) had a failed molecular analysis due to insufficient DNA extraction—29 (32.58%), 6 (6.74%), and 3 (3.37%) were found to have *KRAS* exons 2–4, *NRAS* exons 2–4, and *BRAF v600* mutations, respectively. Moreover, 36 patients were tested for *TP53* and *PIK3CA* mutation; 26 (72.22%); and 5 (13.89%) were found to have these mutations, respectively. These results are largely consistent with previously published literature.

### gDNA Extraction From Biopsy: A Cohort of PROSPECT-C and PROSPECT-R Studies

DNA was extracted from 62 biopsies taken from our prospective PROSPECT-C and PROSPECT-R trials and in 65% of cases; sufficient gDNA for WES was achieved from a single core. Two or three tissue cores were needed to yield sufficient DNA for 27 and 8% of the biopsy time points, respectively (Supplementary Figure 1). When required, utilizing all available tissue cores allowed gDNA extraction rate of 100%. Tumor content was determined for 62 biopsies (75.61%) in the analyzed cohort and was estimated as >20% in 79.03% of cases (Table 1).

**TABLE 1:** DNA extraction and estimated tumor content.

	PROSPECT-C	PROSPECT-R	Total
Number of BL	15	31	46
Number of PD	15	21	36
Number of pairs (BL/PD)	7	19	26
Attempted DNA extraction (total)	30	33	63
Attempted DNA extraction (BL)	15	31	46
Attempted DNA extraction (PD)	15	2	17
Attempted DNA extraction (pairs BL/PD)	7	2	9
DNA yield > 1 μg (total)	27	31	58
DNA yield > 1 μg (BL)	13	29	42
DNA yield > 1 μg (PD)	14	2	16
DNA yield > 1 μg (pairs BL/PD)	6	2	8
Estimated tumor content (total)	30	32	62
≥20%	22	27	49
<20%	8	5	13
Estimated tumor content (BL)	15	31	46
≥20%	11	27	38
<20%	4	4	8
Estimated tumor content (PD)	15	1	16
≥20%	11	0	11
<20%	4	1	5
Estimated tumor content in BL/PD pairs	7	1	8
≥20%	4	0	4
<20%	3	1	4

*BL,baseline; PD, progressive disease.*

### Assessment of Biopsy Tumor Content

All patients entering the PROSPECT trials were tested for *KRAS*/NRAS mutations in the archival tumor biopsy by standard COBAS methodology, as this precluded entry into PROSPECT-C study (2). As a result, all patients entering the PROSPECT-R trial had a cataloged *KRAS*/*NRAS* variant that could be used to investigate the tumor content of the respective biopsy samples (1). Mutation profiles for *KRAS*/*NRAS* have previously been shown to be highly concordant between samples from the same colorectal tumor (9). We therefore estimated the cancer cell content of biopsy samples using Sanger sequencing to detect the likely truncal *KRAS/NRAS* mutations identified previously by clinical sequencing assays. Samples were scored according to the following criteria: “high” tumor content if the variant base was detected at an intensity exceeding or equal to the wild-type base; “medium” if the variant base was detected at >25% of the intensity of the wild-type base; “low” if the variant base was clearly detected above background but at <25% of the intensity of the wild-type base; and “not detected” if the variant base could only be detected within the background noise or not at all (Table 2 and Figure 3). Further cores were extracted and sequenced if the first had low or no detectable tumor content (Table 2). In five cases, an additional core had medium tumor content where the first tested core has low/not detectable tumor content. Out of the 49 samples tested, 39 were scored as medium or high tumor content (Table 2).

**TABLE 2:** Sample tumor content estimated from Sanger sequencing.

	PROSPECT-R
Number of samples sequenced:	49
Number of cores sequenced per sample:	
1 core:	35
2 cores:	10
3 cores:	4
Number of high tumor content cores:	12
Number of medium tumor content cores:	27
Number of low tumor content cores:	6
Number of cores with no detectable tumor content:	22

## Discussion

Tissue biopsies are often considered as the gold standard for diagnostic and research purposes; however, there are many logistical, technical, and ethical challenges in the successful appliance of tissue biopsies in the clinic. To our knowledge, we present the largest dataset of tissue biopsies with a prospective validation cohort demonstrating high tumor yield and ability to perform genomic analysis via image-guided tissue sampling (10, 11).

Biomarker discovery requires validation in prospective clinical trials; however, tissue collection procedures need to be optimized such that the valuable tissue obtained during trials is processed successfully (12, 13). Moreover, even within a resource-friendly environment, molecular profiling studies have often suffered due to inadequacy of samples; failure rates reportedly vary between 15 and 33% (14–17). Keeping these issues in view, we ensured that prebiopsy scans were discussed in person with a radiologist, and only the most amenable lesions were chosen for pretreatment biopsies; experienced radiologists were then able to target multiple cores (6) from the periphery of the chosen lesions. The current study demonstrates that a strong infrastructure and good communication allows high-quality tumor samples to be obtained in a time-efficient manner. The coaxial biopsy technique used at the RM has the advantage of puncturing the capsule of solid organs (liver, kidney, spleen) only once, minimizing the injury to normal tissues, improving patient experience, and at the same time acquiring multiple large cores for diagnosis and molecular analysis. The application of preformed gelatin sponge sealing device at the biopsy tract provides a mechanical matrix that facilitates clotting. Gelfoam pledgets, due to their bulk, surface-acting hemostatic agents, slow the flow of blood, protect the forming clot, and offer a framework for the deposition of the cellular elements of blood, decreasing the risk of major bleeding (18). The grade III hemorrhage in our series was only 0.2%, which compares favorably with the 0.5–2% seen in large series in the literature using Tru-Cut needles with or without coaxial technique (19, 20).

Common concerns about trials mandating research biopsies include the lack of patient understanding about the purpose of such studies and the potential risks associated with additional interventional procedures within the research protocols (21–24). In the case of our PROSPECT-C trial (1, 2), patients included in the study had access to anti-EGFR antibody treatment via the cancer drug fund (CDF) independent of the research biopsy findings, which meant that the research biopsies were of no direct patient benefit. In order to ensure that patients clearly understood the purpose of their participation in PROSPECT-C and other research studies, a prospective patient-based survey at the RM was performed. Remarkably, it showed that most patients who consented to a research biopsy gave an altruistic reason (e.g., to help research and/or others) as to why they agreed to participate (25). A common concern regarding trial-related invasive intervention is procedure-related complications. Notably, the biopsy complication rates in more than 500 patients in our cohort (including patients on PROSPECT studies) were extremely low and compared favorably with published literature (26, 27). The technical reasons for success can be attributed to the use of large gage coaxial needles, which enable multiple tissue cores to be sampled with a single pass. Subsequent application of gelatin foam pledgets via a coaxial cannula at withdrawal effectively seals the biopsy track and minimizes hemorrhage (<1%), thus enabling safe same-day discharge in the majority of patients. This technique, however, needs to be carefully considered in appropriate patients; for example, any attempt to biopsy lung parenchyma would carry a significant risk of the gelfoam pledget deploying in a pulmonary vein resulting in systemic embolus. We, however, acknowledge that the exceptional safety observed in our cohort may not be reproducible in a less resource-friendly environment, as it is highly operator dependent, and thus, clinicians are encouraged to audit their own data when determining the need for requesting tissue biopsies.

Following the safe acquisition of biopsy material, the processing of tumor samples has its own challenges. First, the acquired sample contains a mixture of cancer cells and stroma (connective tissue, blood vessels, and inflammatory cells). It is well established that stromal infiltration may lead to problems in interpreting genomic data (28, 29). In contemporaneous studies conducted at the RM (e.g., FOrMAT study), sample failure rates were high with only 16% of samples showing tumor content of >50% (30). The FOrMAT study collected a range of GI tumor samples including pancreatic cancers, which are more likely to be dominated by inflammatory and stromal cells (31), but it relied on using only FFPE tissues. FFPE tissue has limitations for complex genomic studies, as the DNA yield and quality are affected by the process of fixation and paraffin embedding (32–36). The PROSPECT studies benefited from parallel analysis using both FFPE and fresh frozen tissue, where the former was used for pathological assessment and the latter for molecular characterization and genomic analysis. By utilizing all available tissue cores as required, we achieved a gDNA extraction of >90% and an estimated tumor content of >20% in 87.27% of the cases. These data compare favorably with a recent large-scale study comprising of >10,000 patients, who were subjected to a hybridization-based next-generation sequencing (NGS) panel capable of detecting all-protein coding mutations, copy number alterations, and selected promoter mutations and structural rearrangements (37).

We next took into account the limitations of tumor estimates generated by subjective pathological assessment of tumor morphology and cellularity estimates. Cellularity can be estimated by quantifying the mutant alleles using technologies, such as Sanger or Ion Torrent sequencing, but this requires prior knowledge of the mutation (29, 38); in PROSPECT-R, Sanger sequencing was used to assess tumor content, as *RAS* mutation was a prerequisite for entry into the study. However, only patients with no known *RAS* pathway mutation could participate in PROSPECT-C, so alternative techniques were required for tumor cellularity estimates. An unbiased statistical approach that directly measures tumor content from the DNA sample, therefore, allowed us to take into account factors such as tumor ploidy and intratumor heterogeneity (ITH). This study highlights the safety of tissue biopsies and has significant clinical implications in the management of various malignancies—repeat biopsies should be considered in clinically relevant cases, for example at the time of progression on targeted therapies. Moreover, recent data by our group (1, 2) and others have demonstrated strong concordance between solid and liquid biopsies, and thus, the latter can be considered where a clinically validated panel is available and answers the relevant clinical question.

## Conclusion

Oncologic management and clinical trial participation require accurate histological and molecular characterization. Image-guided biopsies using large gage coaxial needles enable multiple tissue cores to be obtained with a single pass. This increases the probability of diagnostic success for complex molecular analysis. Applying gelatin foam pledgets via the coaxial cannula following biopsy to seal the track reduces hemorrhagic risk and enables safe same-day discharge in the majority of patients. By successfully obtaining sufficient number of tumor tissue samples within prospective trials, such studies can further the understanding of tumor biology and help develop biomarkers of clinical and translational relevance. Ultimately, this will enhance the application of personalized medicine in the clinic.

## Author’s Note

This work was presented in ESMO 2017 and the abstract was published in Annals of Oncology in supplements.

## Data Availability Statement

The accession number for the DNA and RNA sequencing data reported in this manuscript is European Genome-phenome Archive: EGAS00001003367 and EGAD00001004501.

## Ethics Statement

The studies involving human participants were reviewed and approved by The Royal Marsden Research and Ethics Committee. Written informed consent from the participants’ legal guardian/next of kin was not required to participate in this study in accordance with the national legislation and the institutional requirements.

## Author Contributions

All authors contributed to the article and approved the submitted version.

## Conflict of Interest

DC received research funding from Roche, Amgen, Celgene, Sanofi, Merck Serono, Novartis, AstraZeneca, Bayer, Merrimack, and MedImmune. IC has had advisory roles with Merck Serono, Roche, Sanofi Oncology, Bristol Myers Squibb, Eli-Lilly, Novartis, and Gilead Science. He has received research funding from Merck-Serono, Novartis, Roche and Sanofi Oncology, and honoraria from Roche, Sanofi-Oncology, Eli-Lilly, and Taiho. KKh has advisory role with Bayer Oncology group. NV received honoraria from Merck Serono, Bayer, and Eli-Lilly. The remaining authors declare that the research was conducted in the absence of any commercial or financial relationships that could be construed as a potential conflict of interest.

## Footnotes

1 https://openwetware.org/mediawiki/index.php?title=ApE_-_A_Plasmid_Editor_(software_review)&oldid=753142

## References

[1] Khan K Rata M Cunningham D Koh DM Tunariu N Hahne JC Functional imaging and circulating biomarkers of response to regorafenib in treatment-refractory metastatic colorectal cancer patients in a prospective phase II study. *Gut.* (2017) 67:1484–92. 10.1136/gutjnl-2017-314178 28790159 PMC6204951

[2] Khan KH Cunningham D Werner B Vlachogiannis G Spiteri I Heide T Longitudinal liquid biopsy and mathematical modeling of clonal evolution forecast time to treatment failure in the PROSPECT-C phase II colorectal cancer clinical trial. *Cancer Discov.* (2018) 8:1270–85. 10.1158/2159-8290.cd-17-0891 30166348 PMC6380469

[3] Cancer Genome Atlas Network. *TCGA Tissue Sample Requirements: High Quality Requirements Yield High Quality Data.* (2016). Available online at: https://www.cancerresearchuk.org/sites/default/files/smp1_booklet_1.2_-_no_marks.pdf (accessed August 14, 2020).

[4] Hsu MY Pan KT Chen CM Lui KW Chu SY Lin YY CT-guided percutaneous core-needle biopsy of pancreatic masses: comparison of the standard mesenteric/retroperitoneal versus the *trans*-organ approaches. *Clin Radiol.* (2016) 71:507–12. 10.1016/j.crad.2016.02.021 27040800

[5] Olson MC Atwell TD Harmsen WS Konrad A King RL Lin Y Safety and accuracy of percutaneous image-guided core biopsy of the spleen. *AJR Am J Roentgenol.* (2016) 206:655–9. 10.2214/ajr.15.15125 26901024

[6] Ocak S Duplaquet F Jamart J Pirard L Weynand B Delos M Diagnostic accuracy and safety of CT-guided percutaneous transthoracic needle biopsies: 14-Gauge versus 22-gauge needles. *J Vascul Int Radiol JVIR.* (2016) 27:674–81. 10.1016/j.jvir.2016.01.134 27017121

[7] Woolston A Khan K Spain G Barber LJ Griffiths B Gonzalez-Exposito R Genomic and transcriptomic determinants of therapy resistance and immune landscape evolution during Anti-EGFR treatment in colorectal cancer. *Cancer Cell.* (2019) 36:35–50.e9. 10.1016/j.ccell.2019.05.013 31287991 PMC6617392

[8] Vlachogiannis G Hedayat S Vatsiou A Jamin Y Fernandez-Mateos J Khan K Patient-derived organoids model treatment response of metastatic gastrointestinal cancers. *Science (New York N Y).* (2018) 359:920–6. 10.1126/science.aao2774 PMC6112415 29472484

[9] Brannon AR Vakiani E Sylvester BE Scott SN McDermott G Shah RH Comparative sequencing analysis reveals high genomic concordance between matched primary and metastatic colorectal cancer lesions. *Genome Biol.* (2014) 15:454. 10.1186/s13059-014-0454-7 PMC4189196 25164765

[10] Khan K Gonzalez Exposito R Cunningham D Koh DM Woolston A Kouvelakis K Diagnostic accuracy and safety of coaxial core-needle biopsy (CNB) system in a predominanty gastrointestinal oncology patient population, treated at the Royal Marsden (RM) Hospital. *Ann Oncol.* (2017) 28(Suppl. 3):iii6 10.1093/annonc/mdx263.014

[11] Khurum K Gonzalez-Exposito R Cunningham D Koh D Woolston A Barber L Diagnostic accuracy and safety of coaxial core-needle biopsy (CNB) system in Oncology patients treated in a specialist cancer centre with prospective validation within clinical trial data. *medRxiv* (2020). [Preprint]. 10.1101/2020.04.17.20065458 PMC7500492 33014822

[12] Diamandis M White NM Yousef GM. Personalized medicine: marking a new epoch in cancer patient management. *Mol Cancer Res.* (2010) 8:1175–87. 10.1158/1541-7786.mcr-10-0264 20693306

[13] Wistuba II Gelovani JG Jacoby JJ Davis SE Herbst RS. Methodological and practical challenges for personalized cancer therapies. *Nat Rev Clin Oncol.* (2011) 8:135–41. 10.1038/nrclinonc.2011.2 21364686

[14] Ferté C Massard C Ileana E Hollebecque A Lacroix L Ammari S Abstract CT240: Molecular screening for cancer treatment optimization (MOSCATO 01): a prospective molecular triage trial; Interim analysis of 420 patients. *Cancer Res.* (2014) 74:CT2140.

[15] Andre F Bachelot T Commo F Campone M Arnedos M Dieras V Comparative genomic hybridisation array and DNA sequencing to direct treatment of metastatic breast cancer: a multicentre, prospective trial (SAFIR01/UNICANCER). *Lancet Oncol.* (2014) 15:267–74. 10.1016/s1470-2045(13)70611-9 24508104

[16] Meric-Bernstam F Brusco L Shaw K Horombe C Kopetz S Davies MA Feasibility of large-scale genomic testing to facilitate enrollment onto genomically matched clinical trials. *J Clin Oncol Off J Am Soc Clin Oncol.* (2015) 33:2753–62. 10.1200/jco.2014.60.4165 26014291 PMC4550690

[17] Le Tourneau C Delord JP Goncalves A Gavoille C Dubot C Isambert N Molecularly targeted therapy based on tumour molecular profiling versus conventional therapy for advanced cancer (SHIVA): a multicentre, open-label, proof-of-concept, randomised, controlled phase 2 trial. *Lancet Oncol.* (2015) 16:1324–34. 26342236 10.1016/S1470-2045(15)00188-6

[18] Lungren MP Lindquester WS Seidel FG Kothary N Monroe EJ Shivaram G Ultrasound-guided liver biopsy with gelatin sponge pledget tract embolization in infants weighing less than 10 kg. *J Pediatr Gastroenterol Nutrit.* (2016) 63:e147–51. 10.1097/mpg.0000000000001429 27749391

[19] Atwell TD Smith RL Hesley GK Callstrom MR Schleck CD Harmsen WS Incidence of bleeding after 15,181 percutaneous biopsies and the role of aspirin. *AJR Am J Roentgenol.* (2010) 194:784–9. 10.2214/ajr.08.2122 20173160

[20] Lipnik AJ Brown DB. Image-guided percutaneous abdominal mass biopsy: technical and clinical considerations. *Radiol Clin North Am.* (2015) 53: 1049–59. 26321453 10.1016/j.rcl.2015.05.007

[21] Pentz RD Harvey RD White M Farmer ZL Dashevskaya O Chen Z Research biopsies in phase I studies: views and perspectives of participants and investigators. *Irb.* (2012) 34:1–8. PMC3977205 22512092

[22] Olson EM Lin NU Krop IE Winer EP. The ethical use of mandatory research biopsies. *Nat Rev Clin Oncol.* (2011) 8:620–5. 10.1038/nrclinonc.2011.114 21808265 PMC3632075

[23] Saggese M Dua D Simmons E Lemech C Arkenau H. Research biopsies in the context of early phase oncology studies:clinical and ethical considerations. *Oncol Rev.* (2013) 7:e5 10.4081/oncol.2013.232 PMC4419615 25992226

[24] Peppercorn J Shapira I Collyar D Deshields T Lin N Krop I Ethics of mandatory research biopsy for correlative end points within clinical trials in oncology. *J Clin Oncol Off J Am Soc Clin Oncol.* (2010) 28:2635–40. 10.1200/jco.2009.27.2443 20406927 PMC5596502

[25] Moorcraft SY Begum R Cunningham D Peckitt C Baratelli C Gillbanks A Attitudes of patients with gastrointestinal cancers toward research biopsies. *Clin Colorectal Cancer.* (2016) 16:e181–9. 10.1016/j.clcc.2016.09.008 27839727

[26] Tacher V Le Deley MC Hollebecque A Deschamps F Vielh P Hakime A Factors associated with success of image-guided tumour biopsies: results from a prospective molecular triage study (MOSCATO-01). *Eur J Cancer.* (2016) 59:79–89. 10.1016/j.ejca.2016.02.006 27017289

[27] Howlett DC Drinkwater KJ Lawrence D Barter S Nicholson T. Findings of the UK national audit evaluating image-guided or image-assisted liver biopsy. Part II. Minor and major complications and procedure-related mortality. *Radiology.* (2013) 266:226–35. 10.1148/radiol.12120224 23143026

[28] Laird PW. Principles and challenges of genomewide DNA methylation analysis. *Nat Rev Genet.* (2010) 11:191–203. 10.1038/nrg2732 20125086

[29] Song S Nones K Miller D Harliwong I Kassahn KS Pinese M qpure: A tool to estimate tumor cellularity from genome-wide single-nucleotide polymorphism profiles. *PLoS One.* (2012) 7:e45835. 10.1371/journal.pone.0045835 23049875 PMC3457972

[30] Moorcraft SY Gonzalez de Castro D Cunningham D Jones T Walker BA Peckitt C Investigating the feasibility of tumour molecular profiling in gastrointestinal malignancies in routine clinical practice. *Ann Oncol Off J Eur Soc Med Oncol.* (2018) 29:230–6. 10.1093/annonc/mdx631 29361134

[31] Erkan M Reiser-Erkan C Michalski CW Kleeff J. Tumor microenvironment and progression of pancreatic cancer. *Exp Oncol.* (2010) 32:128–31. 21403605

[32] Koshiba M Ogawa K Hamazaki S Sugiyama T Ogawa O Kitajima T. The effect of formalin fixation on DNA and the extraction of high-molecular-weight DNA from fixed and embedded tissues. *Pathol Res Pract.* (1993) 189:66–72. 10.1016/s0344-0338(11)80118-4 8390645

[33] Howat WJ Wilson BA. Tissue fixation and the effect of molecular fixatives on downstream staining procedures. *Methods.* (2014) 70:12–9. 10.1016/j.ymeth.2014.01.022 24561827 PMC4240801

[34] Zsikla V Baumann M Cathomas G. Effect of buffered formalin on amplification of DNA from paraffin wax embedded small biopsies using real-time PCR. *J Clin Pathol.* (2004) 57:654–6. 10.1136/jcp.2003.013961 15166276 PMC1770336

[35] Cree IA Deans Z Ligtenberg MJ Normanno N Edsjo A Rouleau E Guidance for laboratories performing molecular pathology for cancer patients. *J Clin Pathol.* (2014) 67:923–31. 10.1136/jclinpath-2014-202404 25012948 PMC4215286

[36] Cancer Research UK. *CRUK Stratified Medicine Program.* (2016). Available online at: http://wwwcancerresearchukorg/sites/default/files/smp1_booklet_12_-_no_markspdf (accessed May 14, 2016).

[37] Zehir A Benayed R Shah RH Syed A Middha S Kim HR Mutational landscape of metastatic cancer revealed from prospective clinical sequencing of 10,000 patients. *Nat Med.* (2017) 23:703–13. 28481359 10.1038/nm.4333 PMC5461196

[38] Rothberg JM Hinz W Rearick TM Schultz J Mileski W Davey M An integrated semiconductor device enabling non-optical genome sequencing. *Nature.* (2011) 475:348–52. 21776081 10.1038/nature10242

