# Supplementary material for: Diagnostic Accuracy and Safety of Coaxial System in Oncology Patients Treated in a Specialist Cancer Center With Prospective Validation Within Clinical Trial Data
Source: Front Oncol. 2020 Sep 4;10:1634. doi: 10.3389/fonc.2020.01634 (PMC7500492; doi:10.3389/fonc.2020.01634)
Supplement: Supplementary file 1 [file Data_Sheet_1.docx]

**Supplementary data**

**Supplementary Figure 1: Average core requirement for sufficient gDNA extraction**

**
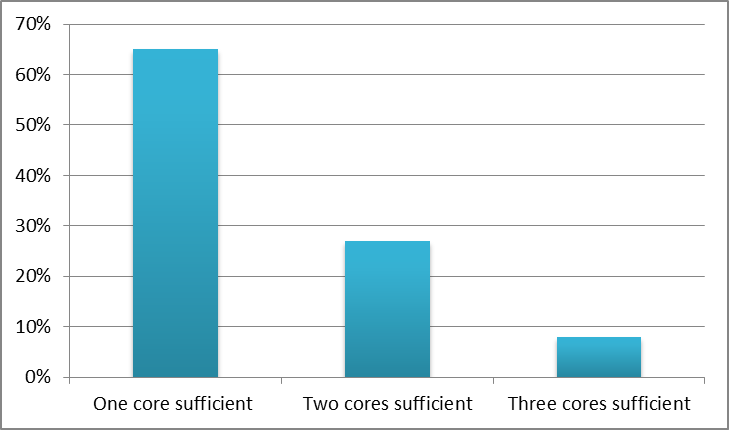
**

**Supplementary Table 1: Sanger Sequencing Primers**

| **Targeted Variant** | **Life Tech. primer pair or primer sequence** | **Annealing temperature** |
| --- | --- | --- |
| KRAS G12/G13 | Hs00532827_CE | 67 **°**C |
| KRAS Q61 | HS00477023_CE | 65 **°**C |
| KRAS K117/A146 | Hs00524892_CE | 65 **°**C |
| NRAS Q61 | Hs00532802_CE | 68 **°**C |
| NRAS G12 | GTGAGGCCGATATTAATCCGG/  TCCGACAAGTGAGAGACAGG | 66 **°**C |

**Supplementary Table 2: Baseline demographics**

Age: Median: 63.18 (IQR: 53.64, 70.31)

| 1. **Gender** | 1. **N** | 1. **%** |
| --- | --- | --- |
| 1. Female | 1. 237 | 1. 51.86 |
| 1. Male | 1. 220 | 1. 48.14 |
|  |  |  |
| 1. Total | 1. 457 | 1. 100 |

| 1. Modality | 1. N | 1. % |
| --- | --- | --- |
| 1. USS | 1. 290 | 1. 63.46 |
| 1. CT | 1. 167 | 1. 36.54 |
|  |  |  |
| 1. Total | 1. 457 | 1. 100 |

| 1. **Primary Tumour** | 1. **Frequency** | 1. **Percentage** |
| --- | --- | --- |
| 1. Upper GI, CUP and hepatobiliary | 1. 89 | 1. 17.05 |
| 1. lymphoproliferative disorders | 1. 43 | 1. 8.24 |
| 1. urological | 1. 60 | 1. 11.49 |
| 1. breast | 1. 45 | 1. 8.62 |
| 1. gynaecological | 1. 38 | 1. 7.28 |
| 1. lower GI | 1. 144 | 1. 27.59 |
| 1. sarcoma | 1. 49 | 1. 9.39 |
| 1. skin | 1. 13 | 1. 2.49 |
| 1. thoracic | 1. 40 | 1. 7.66 |
| 1. other (thyroid) | 1. 1 | 1. 0.19 |
| 1. Total | 1. 522 | 1. 100 |

**Supplementary Table 3A:**

**Liver biopsies (n=284 biopsies from 231 patients)**

Median age of 231 patients: 63.6 (54.8, 70.9)

| 1. **Outcome** | 1. **Frequency** | 1. **Percentage** |
| --- | --- | --- |
| 1. Failure | 1. 14 | 1. 4.9 |
| 1. Success | 1. 267 | 1. 94.0 |
| 1. Yes/N | 1. 3 | 1. 1.1 |
| 1. Total | 1. 284 | 1. 100 |

| 1. Primary Tumour | 1. Frequency | 1. Percentage |
| --- | --- | --- |
| 1. Upper GI, CUP and hepatobiliary | 1. 58 | 1. 20.42 |
| 1. lymphoproliferative disorders | 1. 7 | 1. 2.46 |
| 1. Urological | 1. 5 | 1. 1.76 |
| 1. Breast | 1. 39 | 1. 13.73 |
| 1. Gynaecological | 1. 11 | 1. 3.87 |
| 1. Lower GI | 1. 125 | 1. 44.01 |
| 1. Sarcoma | 1. 6 | 1. 2.11 |
| 1. Skin | 1. 9 | 1. 3.17 |
| 1. Thoracic | 1. 23 | 1. 8.1 |
| 1. Others (thyroid) | 1. 1 | 1. 0.35 |
| 1. Total | 1. 284 | 1. 100 |

| 1. **Reason for delay** | 1. **Frequency** | 1. **Percentage** |
| --- | --- | --- |
| 1. Inpatient | 1. 20 | 1. 46.5 |
| 1. Elective | 1. 7 | 1. 16.3 |
| 1. Late evening | 1. 3 | 1. 7.0 |
| 1. Complications | 1. 13 | 1. 30.2 |
| 1. Total | 1. 43 | 1. 100 |

| 1. **complication type** | 1. **Frequency** | 1. **Percentage** |
| --- | --- | --- |
| 1. Pain | 1. 8 | 1. 57.14 |
| 1. Liver function deranged | 1. 1 | 1. 7.14 |
| 1. Vasovagal | 1. 2 | 1. 14.29 |
| 1. Sepsis | 1. 1 | 1. 7.14 |
| 1. Organ injury | 1. 1 | 1. 7.14 |
| 1. Sepsis | 1. 1 | 1. 7.14 |
| 1. Total | 1. 14 | 1. 100 |

**Supplementary Table 3B:**

**All biopsies except liver (238 biopsies from 228 patients)**

Age; median, (IQR): 63.0 (51.9, 69.4)

| 1. **Outcome** | 1. **Frequency** | 1. **Percentage** |
| --- | --- | --- |
| 1. Failure | 1. 4 | 1. 1.68 |
| 1. Success | 1. 234 | 1. 98.32 |
| 1. Total | 1. 238 | 1. 100 |

| 1. **Site** | 1. **Frequency** | 1. **Percentage** |
| --- | --- | --- |
| 1. Kidney | 1. 35 | 1. 14.71 |
| 1. Abdominal | 1. 44 | 1. 18.49 |
| 1. Pelvic | 1. 60 | 1. 25.21 |
| 1. Lymph Node | 1. 60 | 1. 25.21 |
| 1. Chest wall | 1. 6 | 1. 2.52 |
| 1. Subcutaneous | 1. 1 | 1. 0.42 |
| 1. Breast | 1. 1 | 1. 0.42 |
| 1. Adrenal | 1. 16 | 1. 6.72 |
| 1. Splenic | 1. 5 | 1. 2.1 |
| 1. Pancreatic | 1. 6 | 1. 2.52 |
| 1. Lung | 1. 1 | 1. 0.42 |
| 1. Soft tissue | 1. 2 | 1. 0.84 |
| 1. Oemntal/Peritoneal | 1. 1 | 1. 0.42 |
| 1. Total | 1. 238 | 1. 100 |

| 1. **Primary Tumour** | 1. **Frequency** | 1. **Percentage** |
| --- | --- | --- |
| 1. Upper GI, CUP and hepatobiliary | 1. 31 | 1. 13.03 |
| 1. Lymphoproliferative disorders | 1. 36 | 1. 15.13 |
| 1. Urological | 1. 55 | 1. 23.11 |
| 1. Breast | 1. 6 | 1. 2.52 |
| 1. Gynaecological | 1. 27 | 1. 11.34 |
| 1. Lower GI | 1. 19 | 1. 7.98 |
| 1. Sarcoma | 1. 43 | 1. 18.07 |
| 1. Skin | 1. 4 | 1. 1.68 |
| 1. Thoracic | 1. 17 | 1. 7.14 |
| 1. Total | 1. 238 | 1. 100 |

| 1. **Delay** | 1. **Frequency** | 1. **Percentage** |
| --- | --- | --- |
| 1. Inpatient | 1. 15 | 1. 42.86 |
| 1. Elective | 1. 10 | 1. 28.57 |
| 1. Precaution | 1. 1 | 1. 2.86 |
| 1. Late evening | 1. 5 | 1. 14.29 |
| 1. Complications | 1. 4 | 1. 11.43 |
| 1. Total | 1. 35 | 1. 100 |

| 1. **Complications** | 1. **Frequency** | 1. **Percentage** |
| --- | --- | --- |
| 1. pain | 1. 1 | 1. 25 |
| 1. hematuria | 1. 1 | 1. 25 |
| 1. thrombosis | 1. 1 | 1. 25 |
| 1. hemorrhage | 1. 1 | 1. 25 |
| 1. Total | 1. 4 | 1. 100 |

**Supplementary Table 4:**

**Association of site of biopsy with outcome**

| Outcome | Liver | Other | Total |
| --- | --- | --- | --- |
|  |  |  |  |
| Failure | 14 | 4 | 18 |
|  | 4.98 | 1.68 | 3.47 |
|  |  |  |  |
| Success | 267 | 234 | 501 |
|  | 95.02 | 98.32 | 96.53 |
|  |  |  |  |
| Total | 281 | 238 | 519 |
|  | 100 | 100 | 100 |

p: 0.053 from chi-squared test

**Association of clinical trial with outcome**

|  | Clinical trial | |  |
| --- | --- | --- | --- |
| Outcome | no | yes | Total |
|  |  |  |  |
| Failure | 16 | 2 | 18 |
|  | 4.52 | 1.21 | 3.47 |
|  |  |  |  |
| Success | 338 | 163 | 501 |
|  | 95.48 | 98.79 | 96.53 |
|  |  |  |  |
| Total | 354 | 165 | 519 |
|  | 100 | 100 | 100 |

p: 0.07 from chi-squared test
